# The carbon starvation response of *Aspergillus niger* during submerged cultivation: Insights from the transcriptome and secretome

**DOI:** 10.1186/1471-2164-13-380

**Published:** 2012-08-08

**Authors:** Benjamin M Nitsche, Thomas R Jørgensen, Michiel Akeroyd, Vera Meyer, Arthur FJ Ram

**Affiliations:** 1 Institute of Biology Leiden, Molecular Microbiology and Biotechnology, Leiden University, Sylviusweg 72, 2333 BE Leiden, The Netherlands; 2 Institute of Biotechnology, Applied and Molecular Microbiology, Berlin University of Technology, Gustav-Meyer-Allee 25, Berlin, 13355, Germany; 3 Kluyver Centre for Genomics of Industrial Fermentation, Delft, PO Box 5057, 2600 GA, The Netherlands; 4 Present Address: Novo Nordisk, Protein Expression, 2760 Måløv, Denmark; 5 DSM Biotechnology Center, Beijerinck Laboratory, 2600 MA Delft, PO Box 1, The Netherlands

## Abstract

**Background:**

Filamentous fungi are confronted with changes and limitations of their carbon source during growth in their natural habitats and during industrial applications. To survive life-threatening starvation conditions, carbon from endogenous resources becomes mobilized to fuel maintenance and self-propagation. Key to understand the underlying cellular processes is the system-wide analysis of fungal starvation responses in a temporal and spatial resolution. The knowledge deduced is important for the development of optimized industrial production processes.

**Results:**

This study describes the physiological, morphological and genome-wide transcriptional changes caused by prolonged carbon starvation during submerged batch cultivation of the filamentous fungus *Aspergillus niger*. Bioreactor cultivation supported highly reproducible growth conditions and monitoring of physiological parameters. Changes in hyphal growth and morphology were analyzed at distinct cultivation phases using automated image analysis. The Affymetrix GeneChip platform was used to establish genome-wide transcriptional profiles for three selected time points during prolonged carbon starvation. Compared to the exponential growth transcriptome, about 50% (7,292) of all genes displayed differential gene expression during at least one of the starvation time points. Enrichment analysis of Gene Ontology, Pfam domain and KEGG pathway annotations uncovered autophagy and asexual reproduction as major global transcriptional trends. Induced transcription of genes encoding hydrolytic enzymes was accompanied by increased secretion of hydrolases including chitinases, glucanases, proteases and phospholipases as identified by mass spectrometry.

**Conclusions:**

This study is the first system-wide analysis of the carbon starvation response in a filamentous fungus. Morphological, transcriptomic and secretomic analyses identified key events important for fungal survival and their chronology. The dataset obtained forms a comprehensive framework for further elucidation of the interrelation and interplay of the individual cellular events involved.

## Background

*Aspergillus niger* is a ubiquitous filamentous fungus. According to its saprophytic lifestyle, *A. niger* is capable of secreting large amounts of various plant polysaccharide degrading enzymes. Its naturally high secretion capacity has long been exploited in industrial biotechnology for the production of homologous and heterologous proteins as well as organic acids [1-3]. Many of its products have acquired the GRAS status, meaning that they are generally considered as safe food ingredients [4]. However, besides its positive economic relevance as an industrial workhorse, *A. niger* is a common storage mold causing spoilage of agricultural goods and contamination of food and feedstocks with mycotoxins [5,6]. Although to a much lesser extent than other species of its genus, *A. niger* is an opportunistic pathogen, which can cause invasive aspergillosis in immunocompromised patients [7].

*A. niger* is exclusively known to propagate via an asexual life cycle, which finally leads to the formation of black airborne mitotic spores. Core genes involved in signal transduction and conidiophore development in the model fungus *A. nidulans*[8] have also been identified in *A. niger*[1], suggesting that the regulation of asexual development is conserved. The first step in conidiophore development is the activation of the transcriptional regulator BrlA, which induces the expression of a number of conidiation-specific genes. BrlA expression is autoregulated, resulting in a strong accumulation of its mRNA during asexual development [8]. Although most conidiation studies are performed at a substrate/air interface, conidiation can also be induced in submerged cultures by nutrient limitation such as severe carbon limitation [9-11]. Under these conditions, carbon from endogenous resources becomes mobilized to fuel maintenance and self-propagation. Consequently, the fungal mycelium becomes highly heterogeneous, bearing empty compartments and those that are committed to conidiation [11,12]. While this strategy is beneficial for self-propagation and the exploitation of new substrate sources during saprophytic growth, it may result in a decrease of the active biomass fraction during carbon-limited industrial production processes.

Only a few studies have been conducted to investigate different aspects of aging carbon-limited fungal cultures. As discussed in the review by White *et al.*[12], most of them focus on physiological and morphological aspects. The generic term autolysis has been frequently used to summarize the involved processes. Hallmarks of autolysis are biomass decline, hyphal fragmentation, release of ammonia and increased extracellular hydrolase activity [12]. For different fungal species, the involvement of hydrolases, especially chitinases and glucanases but also proteases has been investigated in great detail [13-15]. An early and strong transcriptional induction in response to carbon starvation was shown in *A. nidualns* for the two hydrolases ChiB and NagA, which have been intensively studied because of their role in the degradation of the cell wall component chitin [16]. In addition to physiological and biochemical hallmarks of aging fungal cultures, several approaches have been developed to quantify the decreasing fraction of active hyphal compartments in aging mycelium by (semi-) automated image analysis [12].

An increasing number of publications highlights the importance of programmed cell death (PCD) in aging fungal cultures [12,17-19]. PCD is generally classified into three types, which are referred to as apoptosis (type I), autophagy (type II) and necrosis (type III) [20]. Their physiological roles are very complex and their relationships are not completely understood. While apoptosis and necrosis are explicitly associated with cell death, autophagy is also a normal physiological process important for cellular homeostasis by lysosomal degradation and recycling. The cellular functions of autophagy have been proposed to exert roles that are both causative of and protective against cell death [20-22].

Improving our understanding of processes induced by carbon starvation and their dynamic interactions is important to further optimize industrial production processes. The aim of this study is to provide a system-wide description of the carbon starvation response of the filamentous fungus *A. niger*. Submerged carbon-limited bioreactor batch cultures were performed and maintained starving up to six days after carbon depletion. In addition to describing the physiology and morphology, we analyzed the secretome and established genome-wide transcriptional profiles for three distinct starvation phases. Besides specifically dissecting expression data for groups of selected genes including proteases, chitinases and glucanases, we performed enrichment analysis to dissect the complex transcriptional changes.

Our investigation shows that carbon starvation in submerged cultures caused complex morphological changes and cellular differentiation including emergence of empty hyphal ghosts, secondary growth of thin non-branching filaments on the expense of older hyphal compartments and formation of conidiating structures. Concomitantly, autophagy and conidiation pathway genes were clearly induced on the transcriptional level. We propose that metabolic adaptation to carbon starvation is mediated by autophagy and that cell death rather than hydrolytic weakening of the fungal cell wall can be considered a hallmark of aging carbon starved *A. niger* cultures.

## Results

### Physiology of carbon starved cultures

**Figure 1:**
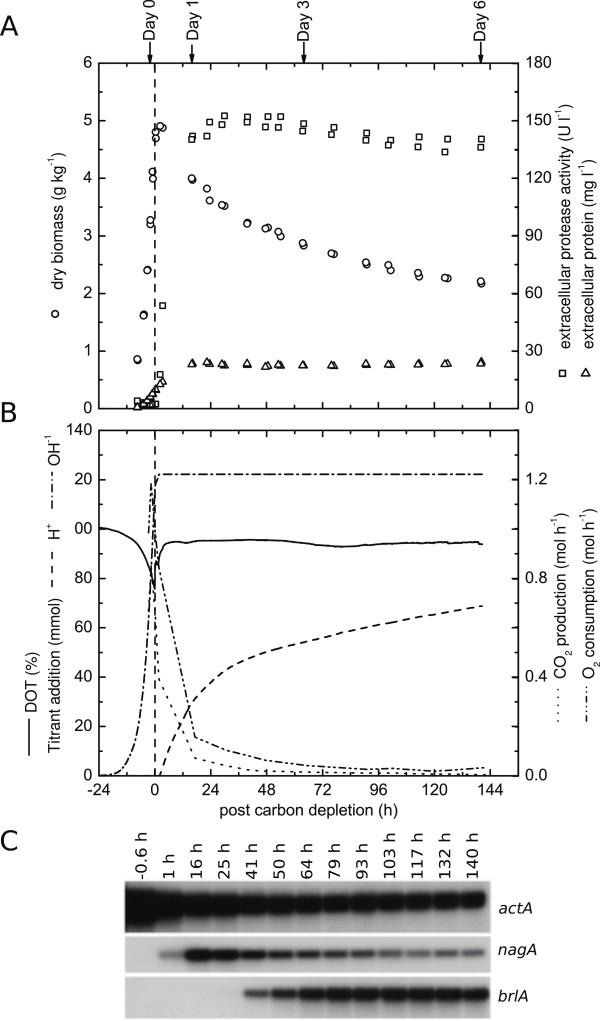
**Physiology and expression profiles of aging carbon limited batch cultures. (A)** Growth curve combined with profiles for extracellular protease activity and extracellular protein concentrations. **(B)** Summary of physiological parameters including dissolved oxygen tension (DOT), titrant addition, O_2_ consumption and CO_2_ production rates. **(C)** Northern analysis for the gamma-actin encoding gene *actA* (An15g00560), the *β*-N-acetylglucosaminidase *nagA* (An09g02240) and the regulator of conidiation *brlA* (An01g10540).

The *A. niger* wild type strain N402 [23] was cultivated under controlled conditions in bioreactors to study its response to carbon starvation during prolonged submerged batch cultivation (Figure 1A and 1B). The defined medium had a pH of 3 and was balanced such, that carbon (maltose) was the growth limiting nutrient. During exponential growth (*μ*_max_ = 0.24h^−1^), pH 3 was maintained by alkaline addition (Figure 1B), which linearly correlated with the biomass accumulation and was previously shown to reflect ammonium uptake during balanced growth on minimal medium [24]. The end of the exponential growth phase was detected by an increase of the dissolved oxygen signal (Figure 1B) and depletion of the carbon source was confirmed by measurements of maltose and glucose concentrations (not shown). The corresponding time point (defined as t=0) was used to synchronize replicate cultures insuring that samples were taken from equivalent physiological phases. The biomass concentration peaked at 5g·kg^−1^ culture broth (Figure 1A).

After maltose was exhausted, pH 3 was maintained by acid addition (Figure 1B). The metabolic activity of the culture decreased in response to the lack of an easily accessible carbon and energy source as indicated by the CO_2_ production and O_2_consumption rates (Figure 1B). Protease activity rapidly increased and was already detected within 3 hours after maltose depletion. During the later starvation phase (up to 140 hours), the protease activity remained constant; however, extracellular protein levels doubled within 16 hours after carbon depletion and remained constant thereafter (Figure 1B). Towards the end of the starvation phase, the cell mass decreased by nearly 60% (Figure 1A). Importantly, CO_2_ and O_2_ levels in the exhaust gas indicated that the cultures were still metabolically active, even 140 hours after depletion of the carbon source (Figure 1B).

### Morphological differentiation during carbon starvation

**Figure 2:**
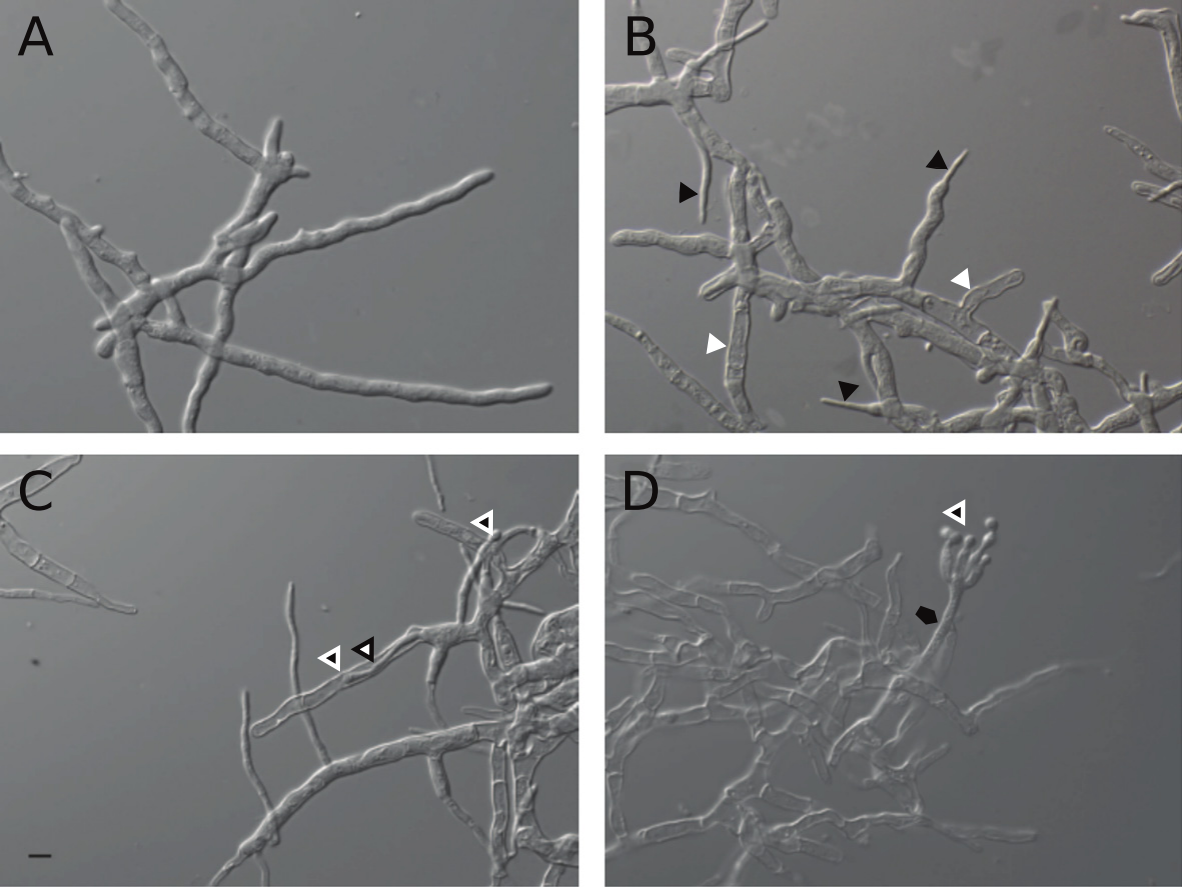
**Hyphal morphology during four distinct cultivation phases. (A)** Intact hyphae from the exponential growth phase with an average diameter of approximately 3 *μ*m. **(B)** 16 hours after carbon depletion empty hyphal compartments emerged (white triangles) and new hyphae with a significantly reduced average diameter of approximately 1 *μ*m appeared (black triangles). **(C)** 60 hours after carbon depletion, the number of empty hyphal compartments increased and thin hyphae elongated in a non-branching manner. First reproductive structures emerged (white-edged triangles). Thin hyphae even grew cryptically inside empty hyphal ghosts (black-edged triangles). **(D)** Even 140 hours after carbon depletion, surviving compartments were present (black pentagon) often bearing morphologically reduced reproductive structures (white-edged triangle). The mycelial network consisted largely of empty hyphal ghosts but hyphal fragmentation was rarely observed. The scale bar refers to 5 *μ*m.

Throughout the entire cultivation, *A. niger* displayed a dispersed morphology. During exponential growth, the mycelium remained intact and no damaged or empty hyphae were observed (Figure 2A). Early after depletion of maltose and onset of starvation, empty hyphal compartments emerged and the diameter of growing hyphae significantly decreased (Figure 2B). Throughout prolonged starvation, the fraction of empty hyphal compartments increased, but the cell wall exoskeleton appeared to remain intact (Figure 2B, 2C and 2D). Fragmented, broken hyphal ghosts were rarely observed. Outgrowing thin filaments emerged, which continued elongating in a non-branching manner. Towards the later starvation phases (60 hours post carbon depletion), morphologically crippled asexual reproductive structures appeared which resembled low-density conidiophores without clearly distinguishable phialides and metulae (Figure 2C and 2D). Even 140 hours after exhaustion of the carbon source, surviving compartments were present, which often showed outgrowing hyphae bearing asexual reproductive structures (Figure 2D). Secondary growth of thin hyphae was even observed within empty hyphal ghosts (Figure 2C).

**Figure 3:**
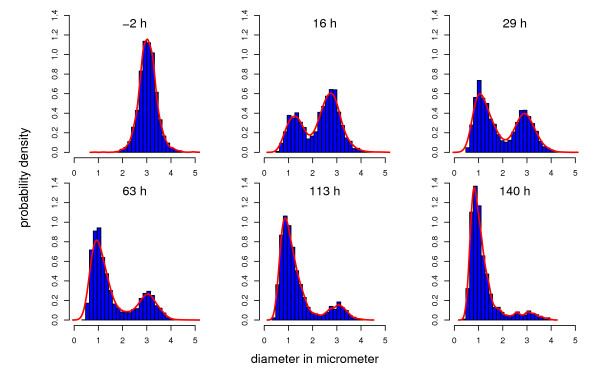
**Hyphal population dynamics.** For six distinct time points, probability density curves of hyphal diameters are shown. 2 hours prior to carbon depletion, a single population of hyphae with a mean diameter of approximately 3 *μ*m was detected. After carbon depletion, a second population with a significantly reduced mean diameter of approximately 1 *μ*m started to emerge. Throughout the course of starvation, the ratio of thin/thick hyphae gradually increased, indicating secondary growth on the expense of dying compartments.

Similar to our results, morphological data from *A. oryzae*[25] indicate a sharp transition between thick and thin compartments (Figure 2B) in response to carbon starvation, suggesting that hyphal diameters can be used to distinguish populations of old and young hyphae formed during primary growth on the supplied carbon source and secondary growth fueled by carbon recycling, respectively. To visualize the transition dynamics from thick (old) to thin (young) hyphae in response to carbon starvation, an image analysis algorithm was developed to analyze hyphal diameter distributions of the cytoplasm filled mycelial fraction. Microscopic pictures from samples of various cultivation time points were analyzed and probability density curves were plotted for the distributions of hyphal diameters (Figure 3). Diameters from exponentially growing hyphae resembled a normal distribution with a mean of approximately 3 *μ*m. In response to carbon starvation, a second population of thinner hyphae with a mean diameter of approximately 1 *μ*m emerged. Throughout the course of starvation, there was a gradual transition from thick (old) to thin (young) hyphae for the cytoplasm filled fraction, suggesting that compartments of older hyphae originating from the exponential growth phase gradually underwent cell death and became empty while a new population of thin hyphae started to grow on the expense of dying compartments.

### Transcriptomic response to carbon starvation

To follow transcriptomic changes during carbon starvation, total RNA was extracted from biomass harvested at different time points during batch cultivation. Although difficulties to isolate intact RNA from aging cultures were reported for *A. nidulans*[26], we could isolate total RNA of high quality from samples up to 140 hours after depletion of the sole carbon source, as assessed by lab on chip quality control (data not shown) and Northern analysis (Figure 1C). Transient expression levels of the gamma-actin encoding gene *actA* (An15g00560), the glycosyl hydrolase *nagA* (An09g02240) and the regulator of asexual sporulation *brlA* (An01g10540) are exemplarily shown in Figure 1C. While *nagA* can be considered an early response gene whose expression peaked 16 hours after exponential growth, *brlA* expression was induced later and remained constant after reaching a plateau at 64 hours of carbon starvation. Expression levels of *actA* decreased considerably after exponential growth but remained constant during later cultivation phases.

**Figure 4:**
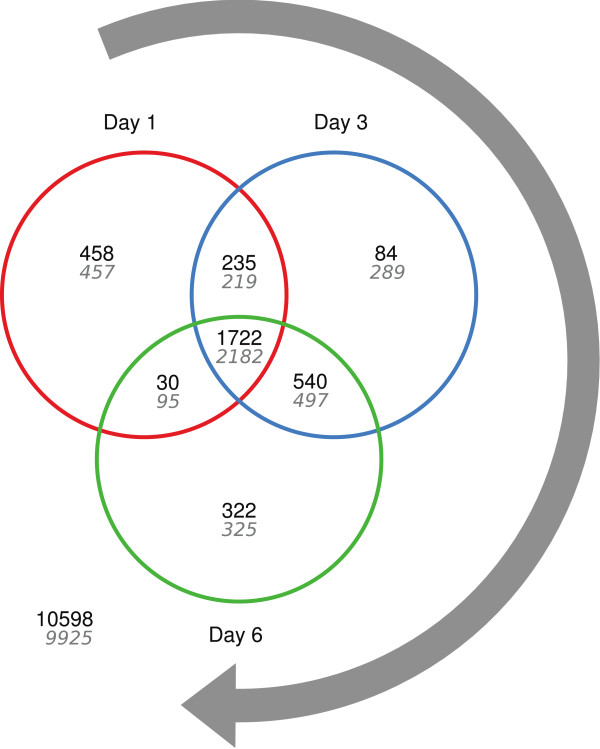
**Venn diagram.** Venn diagram showing numbers of up- and downregulated genes in black and grey, respectively. Differential expression (FDR q-value < 0.005) was assessed by comparison of expression profiles from day 1, 3 and 6 of carbon depletion with expression profiles from the exponential growth phase

RNA samples from four distinct cultivation phases were subjected to genome-wide transcriptional profiling: Exponential growth phase, 16 hours (day 1), 60 hours (day 3) and 140 hours (day 6) post carbon depletion. Differentially expressed genes were identified by a moderated t-test [27] applying a critical FDR q-value of 0.005. Compared to the exponential growth phase, 7,292 of totally 13,989 genes (52%) were identified as differentially expressed during at least one of the starvation time points (Additional file 1). 1,722 genes were conjointly upregulated, whereas 2,182 genes were conjointly downregulated during carbon starvation (Figure 4). Enrichment analyses using Gene Ontology (GO) [28], Pfam domain [29] and Kyoto Encyclopedia of Genes and Genomes (KEGG) [30] pathway annotations were performed to uncover major transcriptional trends. For *A. niger*, all three annotations are based on computational inference. Among them, GO annotation can be considered to have the best quality because it was inferred from the computationally and manually curated GO annotation of the closely related species *A. nidulans*[31].

**Figure 5:**
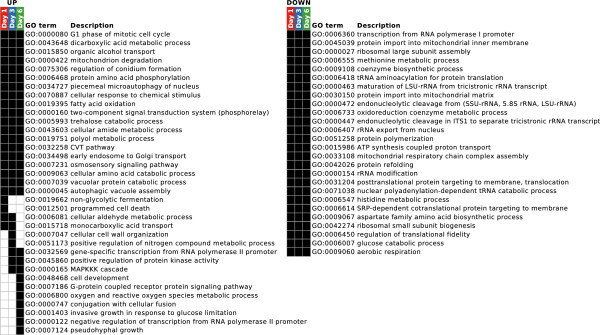
**Summary of GO enrichment results.** Summary of GO enrichment results for the up- and downregulated gene sets of day 1, 3 and 6 of carbon starvation. Statistically significant overrepresentation (FDR < 0.05) is indicated in black.

The GO enrichment results are summarized in Figure 5 (see Additional file 2 for complete GO enrichment results). They cover 20% (668) and 33% (1,334) of all up- and downregulated genes, respectively. Among the genes induced under carbon starvation, common and time-dependent overrepresentation of GO terms was observed. While GO terms related to e.g. catabolic (autophagy, cytoplasm to vacuole targeting (CVT) pathway, fatty acid oxidation and trehalose catabolism) and reproductive (conidiation and mitotic cell cycle) processes were generally enriched, other processes responded in a time-dependent manner constituting early, intermediate or late responses. Among the transiently enriched processes were non-glycolytic fermentation and PCD (day 1), cell wall organization (day 3), regulation of transcription from RNA polymerase II promoter (day 3 and 6) as well as reactive oxygen metabolism (day 6). In contrast to the upregulated genes, the downregulated gene sets did not display any time-dependent differences with respect to the significantly overrepresented GO terms. The commonly downregulated processes included transcription from RNA polymerase I promoter, ribosome biogenesis, translation, secretion and respiration.

Pfam domain and KEGG pathway enrichment results are summarized in the supplemental data (Additional files 3 and 4). Although the three annotations have different sources, structures and levels of complexity, the individual enrichment results confirm each other. Only in a few cases, Pfam domain and KEGG pathway enrichment analyses provided additional information beyond the GO enrichment results. For example, among the upregulated genes at day 1, 3 and 6, those having a putative sugar transporter domain (PF00083) were strongly enriched (49, 40, 39 predicted genes out of 110, respectively). In consideration of the severe carbon limitation, it can be assumed that these predicted sugar transporters comprise high-affinity sugar transporters. Indeed, *mstA* (An12g07450) and *mstF* (An02g00590) encoding two high-affinity sugar/H^+^symporters [32] were significantly upregulated at day 1 and 3 as well as day 1, 3 and 6, respectively. Furthermore, the cytochrome P450 domain (PF00067) was significantly enriched among genes upregulated at day 1. The biochemical roles of the majority of cytochromes P450 are unknown but many are expected to be involved e.g. in the formation of pigments, antioxidants and secondary metabolites [33]. Two of the 44 enriched cytochrome P450 domain proteins are physically associated with distinct (putative) secondary metabolite clusters, of which one is the fumonisin cluster [1]. Obviously, induction of the fumonisin cluster constitutes an early and orchestrated response to carbon starvation. Transcript levels for 11 of the 14 predicted open reading frames were exclusively elevated at day 1, including the putative transcription factor encoded by An01g06900 (Additional file 5).

In addition, PCD-associated genes were specifically overrepresented (q-value < 0.033) during the early adaptive phase at day 1 of carbon starvation. The encoded proteins include two predicted metacaspases (An09g04470, An18g05760) and a Poly(ADP-ribose) polymerase homologue (An18g01170). Four proteins sharing NACHT domains combined with ankyrin or WD40 domain repeats (An11g08920, An01g08000, An01g01380, An07g01930) and three proteins with a NB-ARC domain (An07g01850, An02g07340,An10g00600) were upregulated as well (Additional file 6).

As implied by the enrichment results for both GO and KEGG pathway annotations, carbon starvation coordinately induced the expression of genes involved in autophagic processes. To date, more than 30 autophagy (*atg*) genes have been identified for *Saccharomyces cerevisiae* and other fungi [34,35], 23 of which have a predicted orthologue in *A. niger*. All except one were detected as significantly upregulated during at least one of the starvation time points (see Table 1). The expression level of *atg8* (An07g10020), encoding a lipid-conjugated ubiquitin-like protein that controls the expansion of pre-autophagosomes [36], was the highest among all *atg* genes. At day 3 it reached 75% of the actin expression level during exponential growth. Despite this concerted induction during carbon starvation, it is clearly evident from the expression data that autophagic processes also play an important role during exponential growth, because *atg* gene expression levels ranged from 0.6% (*atg12*: An11g06920) to 24% (*atg8*: An07g10020) when compared with the actin gene expression level (Table 1).

**Table 1:** Expression data of predicted autophagy genes

		**Identifiers**				**Expression**^***a***^					**Fold change**^***b***^				**FDR q-values**^***b***^		
**Gene**	**(Predicted) function**	***S. cerevisiae***	***A. nidulans***	***A. niger***		**Exp**^***d***^	**Day 1**	**Day 3**	**Day 6**		**Day 1**	**Day 3**	**Day 6**		**Day 1**	**Day 3**	**Day 6**
*atg1*	Ser/thr kinase	S000003148	AN1632	An04g03950		1.3	3.2	2.9	2.4		2.4	2.2	1.8		2.5E-07	1.1E-06	1.5E-05
*atg2*	Membrane protein	S000005186	AN5491	An08g10270		3.9	8.2	8.2	7.8		2.1	2.1	2.0		2.8E-08	2.9E-08	6.8E-08
*atg3*	E2-like conjugating enzyme	S000005290	AN11004	An03g04380		1.0	2.9	2.7	2.9		2.9	2.6	2.9		1.8E-08	6.1E-08	1.9E-08
*atg4*	Cysteine protease	S000005167	AN3470	An11g11320		4.6	15.5	16.8	17.3		3.4	3.6	3.7		3.8E-10	2.1E-10	1.6E-10
*atg6*	Subunit of phosphatidylinositol 3-kinase complexes	S000006041	AN10213	An16g07540		1.0	1.0	1.0	0.8		1.0	1.0	0.8		8.3E-01	8.2E-01	5.0E-02
*atg7*	Ubiquitine activating enzyme	S000001214	AN7428	An02g14900^*c*^		1.5	5.6	5.1	3.0		3.8	3.5	2.0		5.7E-08	1.3E-07	6.4E-05
				An02g14910^*c*^		6.5	20.2	18.6	15.7		3.1	2.9	2.4		3.7E-10	1.1E-09	8.3E-09
*atg8*	Autophagosomal membrane protein	S000000174	AN5131	An07g10020		23.8	69.1	75.0	69.7		2.9	3.2	2.9		3.1E-10	1.5E-10	3.1E-10
*atg9*	Transmembrane protein	S000002308	AN3734	An06g01500		1.0	8.0	6.6	5.8		8.0	6.6	5.8		4.1E-11	1.3E-10	3.0E-10
*atg10*	E2-like conjugating enzyme	S000003965	AN10728	An18g06610		1.2	1.4	1.4	1.2		1.1	1.2	1.0		1.0E-01	3.2E-02	7.3E-01
*atg11*	Adapter protein pexophagy and CVT pathway	S000006253	AN2887	An02g07380		2.3	5.2	6.1	4.0		2.2	2.6	1.7		6.2E-08	8.3E-09	5.8E-06
*atg12*	Ubiquitin-like modifier	S000000421	AN1760	An11g06920		0.6	0.8	0.7	0.6		1.4	1.2	1.0		6.4E-03	9.4E-02	7.9E-01
*atg13*	Regulatory subunit of Atg1 signalling complex	S000006389	AN2076	An11g04460		0.8	1.1	1.1	1.2		1.4	1.4	1.6		3.3E-02	2.0E-02	6.6E-03
*atg15*	Vacuolar lipase	S000000664	AN5919	An03g02820		2.6	7.1	6.0	5.5		2.7	2.3	2.1		5.3E-09	4.7E-08	1.9E-07
*atg16*	Atg12-Atg5-Atg16 complex	S000004769	AN0090	An18g02220		1.7	3.5	3.3	2.9		2.1	2.0	1.8		7.1E-05	1.1E-04	6.7E-04
*atg17*	Scaffold protein of Atg1 signalling complex	S000004415	AN6360	An02g04820		1.6	3.5	4.2	4.7		2.2	2.7	3.0		8.6E-09	9.4E-10	2.6E-10
*atg18*	Phosphoinositide binding protein	S000001917	AN0127	An18g03070		2.0	1.8	2.8	2.6		0.9	1.4	1.3		3.7E-01	1.4E-03	4.7E-03
*atg20*	Sorting nexin family member	S000002271	AN6351	An02g01390		4.5	7.7	8.4	9.0		1.7	1.8	2.0		6.1E-06	1.1E-06	3.3E-07
*atg22*	Vacuolar integral membrane protein	S000000543	AN7437	An02g14810		5.7	10.1	9.2	8.9		1.8	1.6	1.6		5.3E-07	4.0E-06	6.8E-06
			AN7591	An09g03630		1.6	1.4	2.0	3.1		0.9	1.3	2.0		3.9E-01	2.0E-02	8.6E-06
			AN5876	An02g03340		1.9	1.3	1.0	1.0		0.7	0.5	0.6		8.7E-04	3.5E-06	1.3E-05
*atg24*	Sorting nexin	S000003573	AN3584	An01g08520		2.8	4.9	4.8	4.9		1.7	1.7	1.7		2.0E-06	3.3E-06	2.3E-06
*atg26*	UDP-glucose:sterol glucosyltransferase	S000004179	AN4601	An07g06610		3.8	10.3	9.8	7.6		2.7	2.6	2.0		2.7E-09	5.8E-09	2.2E-07
*atg27*	Type I membrane protein	S000003714	AN0861	An01g13390		1.1	1.7	1.5	1.5		1.6	1.4	1.4		2.8E-06	1.8E-04	6.8E-05
*atg28*	Coiled-coil protein		AN1701	An04g03260		1.0	1.9	1.9	2.2		1.9	2.0	2.2		5.9E-06	3.7E-06	6.5E-07
*atg29*	Recruitment of proteins to the pre-autophagosomal structure	S000006087	AN4832	An02g13480		1.0	2.5	2.4	2.7		2.4	2.3	2.6		3.2E-08	6.2E-08	1.2E-08

^a^mRNA abundance relative (%) to the gamma-actin (An15g00560) encoding transcript during exponential growth.

^b^Fold changes and FDR q-values for comparisons with transcriptome data from the exponential growth phase.

^c^ORF truncated by contig borders.

^d^Exponential growth phase.

The induction of hydrolases, including proteases and glycosyl hydrolases, has been proposed as a key event in aging fungal cultures [12]. During carbon starvation, glycosyl hydrolases are involved in both the liberation of carbon from fungal cell wall polymers and cell wall remodeling. We identified those upregulated genes that putatively encode glycosyl hydrolases active on fungal cell wall polymers such as chitin, glucan and mannan (Table 2) by mining publicly accessible data [1,37]. The expression profiles allow a general classification into early and late response genes. In agreement with literature [16], the chitinolytic genes *chiB* (An02g07020) and *nagA* (An09g02240) were among the highest induced early response genes. The rapid transient induction of *nagA* as shown by Northern analysis (Figure 1C) exemplarily corroborates the microarray data. In addition to the chitinolytic hydrolases, the group of intensely induced early response hydrolases includes the *α*-glucanase *agnB* (An07g08640), multiple *β*-glucanases and one mannanase. Besides a number of glycosyl hydrolases that were only marginally induced during the later time points, the chitinases *cfcI* (An02g13580) and *ctcB* (An09g05920) showed strong specific induction during the two later time points. It is thus tempting to speculate that *cfcI* and *ctcB* are rather involved in cell wall remodeling during asexual development than liberation of carbon from cell wall polymers.

**Table 2:** Expression and secretome data of predicted glycosyl hydrolases

					**Transcriptomic data**																
					**Expression**^***a***^					**Fold changes**^***b***^				**FDR q-values**^***b***^				**Secretome data**^***c***^			
**Identifier**	**Gene**	**CAZy**	**(Predicted) function**	**SP**^***d***^	**Exp**^***e***^	**Day 1**	**Day 3**	**Day 6**		**Day 1**	**Day 3**	**Day 6**		**Day 1**	**Day 3**	**Day 6**		**Exp**^***e***^	**Day 1**	**Day 3**	**Day 6**
*Chitin*																					
An09g02240	*nagA*	GH20	*β*-1,6-N-acetylglucosaminidase	1	1.1	64	52	48		57.4	46.4	43.5		2.4E-12	5.0E-12	5.6E-12		-	++++	+++++	+++++
An02g07020	*chiB*	GH18	chitinase	0	1.6	73	77	84		45.0	47.2	51.5		3.9E-14	4.1E-14	3.4E-14		-	++	-	-
An01g05360	*cfcD*	GH18	chitinase	0	1.2	10	11	10		8.1	8.9	7.7		2.8E-12	1.9E-12	3.7E-12		-	-	-	-
An01g01920		GH20	*β*-1,6-N-acetylglucosaminidase	1	0.5	2	1	2		3.1	2.5	3.6		5.6E-08	9.3E-07	1.5E-08		-	-	+++	++
An02g02340	*csmB*	GT2	chitin synthase	0	7.7	19	25	22		2.5	3.2	2.9		1.8E-09	1.1E-10	2.9E-10		-	-	-	-
An09g02290	*chsD*	GT2	chitin synthase	0	4.4	10	11	10		2.3	2.6	2.3		5.1E-09	1.4E-09	8.0E-09		-	-	-	-
An09g04010	*chsB*	GT2	chitin synthase	0	7.5	16	16	17		2.1	2.1	2.2		4.1E-08	4.7E-08	1.9E-08		-	-	-	-
An08g05290	*chsG*	GT2	chitin synthase	1	0.5	1	1	0		1.6	1.3	1.0		1.6E-04	1.5E-02	7.5E-01		-	-	-	-
An02g02360	*csmA*	GT2	chitin synthase	0	2.9	5	6	4		1.6	2.0	1.5		2.1E-03	4.9E-05	6.5E-03		-	-	-	-
An02g13580	*cfcI*	GH18	chitinase	1	0.6	1	24	43		2.3	41.6	73.5		1.2E-02	3.9E-09	6.9E-10		-	-	-	-
An09g05920	*ctcB*	GH18	chitinase	1	0.5	1	16	42		1.3	33.8	90.2		1.2E-01	2.1E-11	1.2E-12		-	-	-	+++^∗^
*α*-*glucan*																					
An07g08640	*agnB*	GH71	*α*-glucanase	1	0.5	33	27	6		68.7	57.7	13.4		8.4E-15	1.3E-14	1.2E-12		-	+++	++	-
An15g04760	*agnE*	GH71	*α*-glucanase	1	0.4	0	2	1		1.0	3.6	1.7		7.2E-01	1.5E-08	1.6E-04		-	-	-	-
An09g03100	*agtA*	GH13	*α*-glucan transferase	1	6.2	14	15	18		2.3	2.4	2.8		3.2E-04	1.9E-04	4.0E-05		-	-	-	-
An02g03260	*agsD*	GH13/GT5	*α*-glucan synthase	1	0.4	0	1	1		1.0	2.3	1.5		9.5E-01	1.9E-06	1.4E-03		-	-	-	-
*β*-*glucan*																					
An01g03090		GH81	*β*-glucanase	1	1.1	34	56	57		29.8	49.3	50.5		1.1E-14	4.3E-15	3.7E-15		-	-	-	-
An02g13180		GH55	*β*-glucanase	1	0.4	4	2	3		11.1	6.1	8.1		5.4E-11	1.7E-09	2.9E-10		-	+++	+++^∗^	++^∗^
An01g04560		GH16	*β*-glucanase	1	1.2	10	23	31		8.2	19.8	26.5		1.4E-11	3.3E-13	1.1E-13		-	+++^∗^	++++	++++^∗^
An18g04100		GH28	*β*-glucanase	1	0.8	4	16	17		5.9	21.0	22.8		1.3E-07	2.5E-10	1.8E-10		-	-	-	-
An01g11010	*crhD*	GH16	*β*-glucanase	1	21.3	92	61	44		4.3	2.9	2.1		1.2E-09	6.3E-08	4.6E-06		+++^∗^	+++++	+++++	+++++
An07g04650	*bgtC*	GH17	*β*-glucanase	0	1.4	6	7	6		4.0	5.0	4.3		2.6E-09	5.3E-10	1.6E-09		+^∗^	-	-	-
An01g12450	*bxgA*	GH55	*β*-glucanase	1	7.5	26	23	34		3.5	3.0	4.5		6.4E-08	2.9E-07	7.6E-09		++^∗^	++++	+++++	+++++
An11g01540		GH16	*β*-glucanase	1	1.0	2	1	1		2.1	1.0	0.6		6.2E-06	9.9E-01	2.5E-04		++^∗^	++++	++++	++++^∗^
An10g00400	*gelA*	GH72	*β*-1,3-glucosyl transferase	1	12.6	20	52	50		1.6	4.1	4.0		4.3E-05	1.6E-10	2.1E-10		+++^∗^	++	-	-
An09g00670	*gelD*	GH72	*β*-1,3-glucosyl transferase	1	48.0	75	59	69		1.6	1.2	1.4		3.3E-05	1.5E-02	2.2E-04		+++	++++^∗^	+++++^∗^	+++++
An02g00850		GH16	*β*-glucanase	1	1.5	2	6	9		1.4	3.8	6.1		1.9E-03	1.3E-09	3.4E-11		-	-	-	++
An06g01550	*fksA*	GT48	*β*-1,3-glucan synthase	0	62.6	71	78	91		1.1	1.2	1.4		7.0E-02	2.6E-03	1.7E-05		-	-	-	-
An08g03580	*bgtA*	GH17	*β*-glucanase	1	0.7	1	1	2		1.1	1.9	2.8		4.3E-01	1.5E-05	9.9E-08		-	-	-	-
An02g09050	*gelG*	GH72	*β*-1,3-glucosyl transferase	1	0.6	1	2	6		1.0	3.6	9.0		9.3E-01	2.5E-06	4.6E-09		-	-	-	-
An16g02850		GH16	*β*-glucanase	1	1.4	1	5	3		0.8	3.6	2.3		1.3E-01	4.8E-09	7.7E-07		-	-	-	-
An06g01530		GH17	*β*-glucanase	1	0.6	1	1	3		0.8	2.3	4.4		2.5E-02	1.9E-07	2.3E-10		-	-	-	-
An02g03980	*kslA*	GH16	*β*-glucanase	0	0.5	0	1	2		0.8	2.4	4.7		4.2E-03	1.5E-08	2.0E-11		-	-	-	-
*Mannan*																					
An07g07700		GH76	*α*-1,6-mannanase	1	1.4	30	35	27		20.8	24.7	18.8		7.1E-13	4.5E-13	1.1E-12		-	+++	-	-
An01g06500	*dfgD*	GH76	*α*-1,6-mannanase	1	0.5	3	5	6		5.8	8.4	10.9		2.3E-10	2.9E-11	7.0E-12		-	-	-	-
An14g03520	*dfgC*	GH76	*α*-1,6-mannanase	1	3.6	7	6	7		1.9	1.8	1.9		1.1E-07	5.8E-07	1.9E-07		-	++^∗^	+++^∗^	-
An04g09650		GH76	*α*-1,6-mannanase	1	0.3	1	0	0		1.8	1.3	1.3		4.0E-05	3.4E-02	2.3E-02		-	+++^∗^	+++^∗^	+++
An02g02660	*dfgG*	GH76	*α*-1,6-mannanase	1	0.8	1	1	1		1.6	1.4	1.1		4.7E-03	3.5E-02	4.7E-01		-	-	-	-
An18g01410	*dfgA*	GH76	*α*-1,6-mannanase	1	0.7	1	1	2		0.8	1.5	2.7		2.4E-01	4.1E-03	1.9E-06		-	-	-	-

^a^mRNA abundance relative (%) to the gamma-actin (An15g00560) encoding transcript during exponential growth.

^b^Fold changes and FDR q-values for comparisons with transcriptome data from the exponential growth phas.

^c^Protein abundance in filtrates: (-) not detected; (+) < 5 ng ml^−1^; (++) < 50 ng ml^−1^; (+++) < 250 ng ml^−1^; (++++) < 1 *μ*g ml^−1^; (+++++) < 4 *μ*g ml^−1^; (++++++) > 4 *μ*g ml^−1^; (^∗^) biological and/or technical relative standard deviation above 100 and 50, respectively.

^d^Signal peptide sequence prediction [43].

^e^Exponential growth phase.

The second group of hydrolases, namely proteases, fulfills diverse physiological functions ranging from signaling to nutrient recycling. In accordance to the rapidly increasing extracellular protease activity after carbon depletion (Figure 1A), an early transcriptional induction of extracellular proteases was observed (Table 3). Compared to exponential growth, the expression levels of the two major secreted proteases *pepA* and *pepB*[38] were increased by more than 130 fold at day 1. Additionally, roughly 20 further predicted secreted proteases were induced during carbon starvation with transcript level changes ranging from 2 to 40. In agreement, expression of the main transcriptional regulator of proteases PrtT [39] was strongly upregulated. Furthermore, transcript levels of about 20 proteases lacking predicted signal peptide sequences were identified as significantly elevated (Table 3), suggesting considerable intracellular proteolytic activities during carbon starvation.

**Table 3:** Expression and secretome data of predicted protein hydrolases

				**Transcriptomic data**																
				**Expression**^**a**^					**Fold changes**^**b**^				**FDR q-values**^**b**^				**Secretome data**^**c**^			
**Identifier**	**Gene**	**(Predicted) function**		**Exp**^*d*^	**Day 1**	**Day 3**	**Day 6**		**Day 1**	**Day 3**	**Day 6**		**Day 1**	**Day 3**	**Day 6**		**Exp**^***d***^	**Day 1**	**Day 3**	**Day 6**
*SP*^*e*^*present*																				
An01g00530	*pepB*	A4 family peptidase		0.6	84.9	32.5	113.9		148.1	56.7	198.8		1.4E-16	9.1E-16	5.9E-17		-	-	-	-
An14g04710	*pepA*	Aspartyl protease		1.4	180.1	34.7	70.5		130.2	25.1	51.0		1.9E-13	2.2E-11	2.2E-12		+++^∗^	++++++	++++++	++++++
An01g01750		Subtilisin-like serine protease		1.1	45.8	9.8	8.9		40.0	8.6	7.7		2.3E-14	9.5E-12	1.5E-11		-	+++	++++	+++
An02g01550		Secreted serine protease		2.4	63.2	25.3	6.0		26.0	10.4	2.5		8.6E-10	4.8E-08	9.8E-04		++^∗^	++++	++++^∗^	-
An08g04640		Lysosomal pepstatininsensitive protease		1.1	16.2	8.0	10.6		14.3	7.1	9.3		4.0E-12	1.5E-10	3.2E-11		+^∗^	++++	+++++	++++
An06g00190		Lysosomal pepstatininsensitive protease		2.3	31.5	27.2	25.9		13.4	11.6	11.0		1.1E-10	2.3E-10	2.8E-10		+++^∗^	++++	+++++	++++
An03g01010		Lysosomal pepstatininsensitive protease		1.2	14.9	3.2	4.2		12.7	2.7	3.6		1.2E-09	4.8E-05	3.6E-06		+^∗^	+++^∗^	+++^∗^	+++^∗^
An02g04690		Serine-typecarboxypeptidase I		6.4	64.7	31.7	34.9		10.1	4.9	5.4		9.7E-10	8.4E-08	4.2E-08		++	++++^∗^	+++++	+++++
An12g05960		Dipeptidyl peptidase II		1.0	9.2	4.1	2.7		9.0	4.0	2.6		2.8E-11	7.3E-09	4.78-07		-	+++^∗^	++++	++++
An07g08030	*pepF*	Serine carboxypeptidase		0.8	7.0	4.2	6.3		8.6	5.2	7.7		1.2E-11	2.7E-10	2.2E-11		-	++^∗^	+++	+++^∗^
An11g06350		Carboxypeptidase		1.1	8.3	3.6	1.7		7.9	3.4	1.6		5.0E-11	2.1E-08	5.0E-04		-	-	-	-
An09g03780	*pepD*	Subtilisin-like serineprotease		0.5	3.0	0.6	0.7		5.8	1.1	1.4		2.2E-11	1.6E-01	2.4E-03		-	-	-	-
An12g03300		Aspartic protease		0.7	3.4	0.5	0.4		5.0	0.7	0.7		1.1E-08	1.2E-02	1.0E-02		++^∗^	+++		
An15g06280		Aspartic proteinase[truncated ORF]		5.8	25.2	33.2	22.4		4.3	5.7	3.8		4.1E-09	6.1E-10	1.2E-08		++^∗^	+++^∗^	++++^∗^	++++^∗^
An16g09010		Carboxypeptidase I[putative frameshift]		0.6	2.2	3.6	4.7		4.0	6.4	8.4		2.5E-09	8.8E-11	1.8E-11		-	-	+++^∗^	+++
An14g00620		Aminopeptidase		5.3	20.4	14.7	14.3		3.8	2.8	2.7		4.6E-09	1.3E-07	1.8E-07		-	-	-	-
An07g03880	*pepC*	Serine proteinase		31.1	111.2	101.5	91.5		3.6	3.3	2.9		8.5E-12	2.2E-11	6.3E-11		++^∗^	-	-	-
An07g10060		Proteinase B inhibitor		4.2	13.1	17.0	19.5		3.1	4.1	4.7		1.1E-08	1.1E-09	3.5E-10		+++^∗^	-	-	-
An02g07210	*pepE*	Aspartic protease		30.4	83.9	57.8	57.2		2.8	1.9	1.9		1.4E-10	3.3E-08	3.9E-08		-	++++	+++^∗^	++^∗^
An15g07700		Aspergillopepsin IIprecursor		1.3	3.5	3.3	4.4		2.7	2.6	3.4		3.5E-07	5.2E-07	3.1E-08		-	-	-	-
An18g01320		Extracellular proteaseprecursor		21.9	54.8	14.7	4.9		2.5	0.7	0.2		1.8E-05	1.5E-02	6.8E-08		+++^∗^	++++	+++++^∗^	++++
An02g13740		Gly-X carboxypeptidaseprecursor		1.9	4.4	4.2	4.4		2.3	2.2	2.4		9.5E-09	2.1E-08	8.6E-09		-	-	-	-
An03g01660		Vacuolar aminopeptidase Y		9.1	19.2	17.4	17.5		2.1	1.9	1.9		3.1E-09	1.9E-08	1.5E-08		++^∗^	-	-	-
An08g08750	*cpY*	Carboxypeptidase		33.8	67.9	53.8	51.3		2.0	1.6	1.5		1.0E-08	1.3E-06	4.2E-06		++^∗^	-	-	-
An14g03250		Aspergillopepsin II		0.7	1.2	0.9	1.6		1.7	1.3	2.2		2.5E-04	3.0E-02	3.1E-06		-	+++^∗^	-	-
An07g10410		Metalloprotease		1.2	1.3	7.5	16.1		1.1	6.5	13.9		2.8E-01	8.3E-11	1.6E-12		-	-	-	-
*SP*^*e*^*absent*																				
An01g00370		Aspergillopepsin		0.8	89.0	16.8	10.0		110.2	20.8	12.3		3.6E-14	4.9E-12	3.9E-11		++^∗^	+++++	+++++	+++++
An02g00090		Prolidase		0.4	20.4	22.7	7.6		53.2	59.4	19.8		1.2E-14	1.0E-14	1.9E-13		-	-	-	-
An14g02080		Prolidase		1.8	18.0	6.8	4.1		9.7	3.7	2.2		4.4E-09	2.8E-06	3.8E-04		-	-	-	-
An09g02830		Acylaminoacyl-peptidase		4.3	39.7	18.0	9.9		9.2	4.2	2.3		1.8E-12	3.0E-10	1.6E-07		-	-	-	-
An17g00390		Aminopeptidase		4.6	27.7	10.2	7.4		6.0	2.2	1.6		3.2E-11	5.1E-07	1.2E-04		-	-	-	-
An18g03980		Glutamatecarboxypeptidase II		3.2	19.0	13.8	13.7		5.9	4.3	4.3		3.3E-12	3.3E-11	3.4E-11		-	-	-	-
An01g01720		Bleomycin hydrolase		0.6	3.3	1.4	1.0		5.4	2.3	1.7		1.6E-10	8.4E-07	1.0E-04		-	-	-	-
An07g06490		Insulin-degrading enzyme		0.4	2.1	1.1	1.1		5.2	2.7	2.7		8.5E-11	2.9E-08	3.7E-08		-	-	-	-
An11g05920		Prolidase		0.9	4.6	1.5	1.3		5.0	1.6	1.4		5.2E-08	9.1E-03	5.3E-02		-	-	-	-
An11g02950		Calpain family cysteine protease		0.9	3.6	3.2	1.7		4.3	3.7	2.0		9.8E-10	3.4E-09	6.0E-06		-	-	-	-
An01g14920		Metallopeptidase		0.5	1.8	1.3	1.1		3.9	2.8	2.3		1.1E-09	4.0E-08	4.1E-07		-	-	-	-
An11g01970		Pyroglutamyl peptidase		2.1	8.2	6.0	6.8		3.9	2.8	3.2		1.0E-11	2.4E-10	6.2E-11		-	-	-	-
An14g01530		Subtilisin-like serine proteases		0.5	2.0	1.1	0.7		3.7	2.1	1.3		9.9E-09	7.0E-06	2.6E-02		-	-	-	-
An09g06800		Leucyl aminopeptidase		10.9	40.3	37.7	33.4		3.7	3.5	3.1		1.3E-11	2.5E-11	8.2E-11		-	-	-	-
An12g01820		Ubiquitin carboxyl-terminal hydrolase		0.8	2.6	1.7	1.1		3.4	2.2	1.5		8.1E-10	2.0E-07	4.4E-04		-	-	-	-
An01g08470		Ubiquitin carboxyl-terminal hydrolase		1.8	5.9	7.5	7.4		3.3	4.2	4.1		6.1E-10	8.3E-11	9.6E-11		-	-	-	-
An04g00410		Dipeptidyl peptidase III		17.0	51.9	37.8	29.6		3.1	2.2	1.7		9.6E-10	5.6E-08	3.4E-06		-	-	-	-
An16g08150		Dipeptidyl-peptidase V		10.5	26.0	17.4	17.2		2.5	1.7	1.6		4.3E-09	4.1E-06	4.9E-06		-	-	-	-
An18g02980		Endopeptidase		4.6	10.4	8.3	8.2		2.3	1.8	1.8		4.0E-09	2.0E-07	2.6E-07		-	-	-	-
An04g06940	*prtT*	Transcriptional activator of proteases		5.4	58.4	35.2	24.9		10.8	6.5	4.6		5.7E-12	9.8E-11	1.0E-09		-	-	-	-

^a^mRNA abundance relative (%) to the gamma-actin (An15g00560) encoding transcript during exponential growth.

^b^Fold changes and FDR q-values for comparisons with transcriptome data from the exponential growth phase.

^c^Protein abundance in filtrates: (-) not detected; (+) < 5 ng ml^−1^; (++) < 50 ng ml^−1^; (+++) < 250 ng ml^−1^; (++++) < 1 *μ*g ml^−1^; (+++++) < 4 *μ*g ml^−1^; (++++++) > 4 *μ*g ml^−1^; (^∗^) biological and/or technical relative standard deviation above 100 and 50, respectively.

^d^Exponential growth phase.

^e^Signal peptide sequence prediction [43].

Northern (Figure 1C), microscopic (Figure 2C and 2D) and GO enrichment (Figure 5) analyses clearly indicated that conidiation is one of the main responses provoked by carbon starvation. Transcriptomic data of a subset of genes predicted to be involved in asexual development in *Aspergillus* are shown in Table 4. Expression profiles of orthologous genes belonging to the two core regulatory pathways identified in *A. nidulans*, STUNTED (*stuA*→*wetA*) and BRISTLE (*brlA*→*abaA*→*wetA*) [40,41] suggest conservation of these regulatory pathways between the two *Aspergilli*. Whereas the first pathway is induced early upon achievement of asexual developmental competence (day 1), induction of the latter pathway is delayed (day 3). Among the fluffy genes *flbA-E* encoding upstream regulators of BrlA [8], only *flbC* and *flbD* were clearly induced. Remarkably, although only little asexual differentiation occurred, hydrophobins were among the most intensely induced genes (Table 4). In a global ranking based on highest expression levels at day 6, the three predicted hydrophobins encoded by An03g02400, An08g09880 and An03g02360 were at positions one, five and six, respectively. In agreement, conidial pigmentation genes including *olvA* were strongly induced (Table 4).

**Table 4:** Expression data of predicted conidiation genes

			**Expression**^***a***^					**Fold changes**^***b***^				**FDR q-values**^***b***^		
**ORF**	**Gene**	**(Predicted) function**	**Exp**^***c***^	**Day 1**	**Day 3**	**Day 6**		**Day 1**	**Day 3**	**Day 6**		**Day 1**	**Day 3**	**Day 6**
*Fluffy genes*														
An14g03390	*fluG*	Synthesis of small extracellular factor	1.9	1.0	1.3	1.8		0.5	0.7	0.9		3.1E-08	3.7E-05	3.0E-01
An02g03160	*flbA*	Regulator of G-protein signalling	0.9	0.8	0.9	1.2		0.9	1.0	1.4		3.3E-01	7.9E-01	5.4E-04
An15g03710	*flbB*	Transcription factor	2.6	2.5	2.1	2.5		1.0	0.8	1.0		6.6E-01	3.6E-02	7.8E-01
An02g05420	*flbC*	Transcription factor	1.1	2.4	4.5	8.2		2.1	4.0	7.2		4.0E-05	3.3E-08	5.1E-10
An01g04830	*flbD*	Transcription factor	1.3	8.2	6.3	9.0		6.3	4.8	6.9		1.0E-09	7.3E-09	6.2E-10
An08g07210	*flbE*	Activator functionally associated with FlbB	1.9	2.0	3.4	4.5		1.1	1.8	2.4		3.7E-01	2.3E-06	2.7E-08
An08g06130	*fadA*	Heterotrimeric G-protein *α*-subunit	15.2	18.8	18.4	19.8		1.2	1.2	1.3		2.0E-03	4.0E-03	2.6E-04
*Conidiophore development*														
An01g10540	*brlA*	Transcription factor	0.4	0.4	13.4	17.0		1.1	37.0	46.8		8.5E-01	3.5E-10	1.7E-10
An01g03750	*abaA*	Transcription factor	1.1	1.0	14.1	31.9		0.9	12.6	28.6		1.7E-01	3.1E-12	1.3E-13
An01g08900	*wetA*	Transcription factor	0.8	0.8	1.5	4.2		0.9	1.8	5.1		4.6E-01	5.1E-06	3.2E-11
An02g02150	*medA*	Transcription factor	1.6	3.6	3.8	2.5		2.2	2.3	1.5		6.2E-08	2.8E-08	7.1E-05
An05g00480	*stuA*	Transcription factor	12.5	28.0	49.7	48.7		2.2	4.0	3.9		5.3E-07	1.0E-09	1.2E-09
*Pigmentation genes and hydrophobins*														
An03g02400	*hypC*	Hydrophobin	0.6	0.6	261.2	333.0		1.1	436.9	557.1		8.7E-01	4.0E-10	2.4E-10
An08g09880		Hydrophobin	0.5	0.5	180.5	229.9		1.1	360.5	459.1		8.9E-01	5.0E-10	3.0E-10
An03g02360	*hypB*	Hydrophobin	0.5	0.4	162.4	214.5		0.9	345.1	455.8		7.3E-01	5.8E-10	3.2E-10
An14g05350	*olvA*	Hydrolase involved in pigmentation	1.6	11.1	153.3	205.4		6.8	94.2	126.2		8.2E-07	3.7E-11	1.8E-11
An01g13660	*yA*	Laccase invovled in pigmenation	0.8	1.0	46.1	20.6		1.2	56.5	25.3		3.6E-01	2.3E-11	3.0E-10
An14g05370	*brnA*	Multicopper oxidase involved in pigmentation	0.7	0.5	33.0	39.9		0.7	46.3	55.9		3.1E-01	1.8E-09	9.6E-10
An01g10940	*hypA*	Hydrophobin	0.4	0.4	12.3	15.0		0.8	29.3	35.6		5.1E-01	1.3E-09	6.4E-10
An07g03340	*hypE*	Hydrophobin	1.9	2.4	26.9	62.3		1.3	14.4	33.4		3.9E-01	9.6E-08	3.8E-09
An09g05730	*fwnA*	Polyketide synthase involved in pigmentation	2.4	1.2	18.8	36.0		0.5	7.7	14.7		2.6E-03	3.6E-08	1.4E-09
An09g05530	*hypG*	Hydrophobin	0.5	0.4	0.8	1.7		0.8	1.5	3.3		2.4E-02	4.4E-04	2.1E-09

^a^mRNA abundance relative (%) to the gamma-actin (An15g00560) encoding transcript during exponential growth.

^b^Fold changes and FDR q-values for comparisons with transcriptome data from the exponential growth phase.

^c^Exponential growth phase.

### Secretomic response to carbon starvation

To identify extracellular hydrolases secreted at various cultivation time points, mass spectrometric analyses of tryptically digested proteins precipitated from culture filtrates were performed. Neither chitin, *α*-glucan nor mannan active hydrolases were detected in the culture broth during exponential growth (Table 2). In agreement to its high transcript levels during carbon starvation, NagA (An09g02240) was the most abundant extracellular hydrolase involved in chitin degradation at day 1, 3 and 6. However, the chitinase ChiB was, in contrast to its strong transcriptional upregulation, only marginally detected in filtrates at day 1. Both observations correspond well to the presence and absence of predicted signal peptide sequences for NagA and ChiB, respectively. Interestingly, ChiB of *A. niger* showed only low extracellular abundance, whereas the *A. nidulans* ChiB (AN4871) was identified as the major extracellular autolytic chitinase during carbon starvation [16]. The absence of ChiB in the culture broth of *A. niger* could explain why hyphal ghosts remained intact but were reported to fragment in aging cultures of *A. nidulans*[42]. In concordance with its expression profile, the *α*-glucanase AgnB (An07g08640) was detected extracellularly at day 1 and 3. While GelD (An09g00670) was the only reliably detected *β*-glucanase during exponential growth, various *β*-glucanases with predicted signal peptide sequences were detected at day 1, 3 and 6 of carbon starvation. Among the predicted mannanases, only An04g09650 was reliably detected in filtrates at later time points (day 3 and 6).

In agreement with increasing extracellular protease activity and expression profiles, a number of proteases with predicted signal peptide sequences were identified in culture filtrates of day 1, 3 and 6. Among them, PepA (An14g04710), the major extracellular protease [38], was most abundant. However, although PepB (An01g00530) has a predicted signal peptide sequence and showed strong transcriptional induction, it was not detected in culture filtrates. Transcriptionally induced proteases lacking predicted signal peptide sequences were not detected in culture filtrates, with the only exception of An01g00370. Similar results have been previously reported for *A. niger* by Braaksma *et al.*[43], who proposed that the high extracellular abundance of An01g00370 is likely a result of non-classical secretion rather than lysis.

The secretome during starvation conditions was clearly enriched by an additional group of proteins with strong similarity to phospholipases. Together the four putative phospholipases, An16g01880, An09g02180, An01g14940 and An02g13220 constituted on average about 7% of all detected extracellular proteins during day 1, 3 and 6. All except An02g13220 were transcriptionally induced during carbon starvation. This high abundance of predicted phospholipases during carbon starvation might be indicative for a role of membrane lipids as alternative carbon source during secondary growth. The complete list of identified extracellular proteins is given in Additional file 7.

## Discussion

The present study is the first system-wide description of the carbon starvation response in a filamentous fungus. The application of bioreactor technology allowed highly reproducible culture conditions and physiological synchronization of replicate batch cultures. The use of minimal medium with maltose as the sole limiting nutrient, constant pH, sufficient aeration and homogeneously dispersed mycelial biomass reduced biological and technical variations to a minimum and allowed us to highlight those differences in gene expression, which were in direct relation to carbon starvation.

Submerged growth is fundamentally different from the natural fungal life style. Fungi experience spatio-temporal gradients of various ambient factors such as nutrients, temperature and pH in their natural habitats. These gradients lead to heterogenity within the fungal colony. Several studies have investigated this heterogeneity during growth on agar plates and have characterized differential concentric zones with respect to gene expression and protein secretion [44-46]. Recently, this heterogeneity has even been shown for microcolonies (pellets) in liquid shaken cultures of *A. niger*[47]. In an ideally mixed bioreactor, all dispersed hyphae experience identical environmental conditions and temporal profiles can be monitored and controlled by process parameters. Accordingly, many evolutionary acquired traits contributing to the natural fungal life style such as the formation of substrate exploring hyphae, secretion of certain hydrolases, cell death and conidiation are dispensable during industrial processes and might even negatively affect production yields.

**Figure 6:**
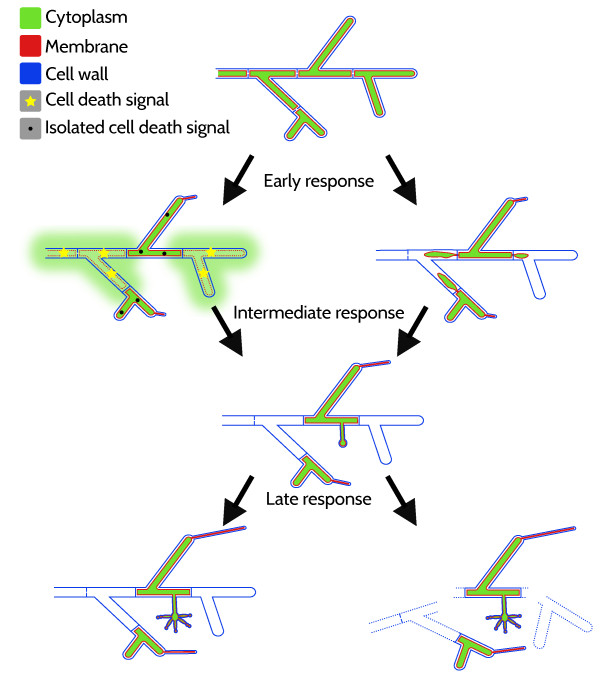
**Model for the carbon starvation response in *A. niger *Schematic representation of major early, intermediate and late processes during prolonged submerged carbon starvation.** During the early phase of starvation, secondary growth fueled by carbon recycling is initiated as characterized by the formation of thin hyphae. Two mechanisms resulting in empty hyphal compartments are depicted. On the left side, apoptotic/necrotic signals lead to cell death of compartments. Cytoplasmic content leaks into the culture broth. Surviving compartments are protected by autophagic processes isolating/inactivating cell death signals. On the right side, endogenous recycling of neighboring compartments by autophagic processes leads to the formation of empty hyphal ghosts. Cytoplasmic content does not leak into the culture broth. During the intermediate phase, earlier processes continue and first reproductive structures emerge. Towards later phase, these processes proceed resulting in few surviving compartments often bearing reproductive structures and elongating thin hyphae. Depending on strain (e.g. *Δ*creA) and cultivation conditions (e.g. elevated pH), a largely empty non fragmented mycelial network remains (left side) or fragmentation of empty hyphal ghosts occurs by hydrolytic weakening of cell walls (right side).

In this study *A. niger* showed general hallmarks of autolysis [12] during prolonged carbon starvation. However, in contrast to *A. nidulans*[42], *A. niger* hyphae did not undergo substantial fragmentation. While an increasing number of hyphal compartments became empty after carbon depletion, microscopic analysis showed that hyphal cell wall skeletons remained mainly intact. Thus disintegration of aging mycelia appears rather to be initiated by intracellular activities such as cell death and/or endogenous recycling of neighboring compartments leading to empty hyphal ghosts than by extracellular hydrolysis of fungal cell walls (Figure 6). This assumption is supported by studies in *A. nidulans*, where autolytic fragmentation of hyphae and cell death were described as simultaneous but independently regulated processes [48]. While deletion of the major carbon catabolite repressor CreA in *A. nidulans* resulted in increased hydrolase activities and mycelial fragmentation during carbon starvation, the viability of *A. nidulans* was not affected [26]. Consistently, we observed hyphal fragmentation and enhanced biomass decline in bioreactor cultures during the starvation phase only when the pH control was switched-off leading to an elevated pH of approximately 5.8 towards the end of cultivation (Nitsche *et al.* unpublished data). We thus propose that hydrolytic weakening of the fungal cell wall and hyphal fragmentation is a secondary effect, which occurs after initial cell death events and only under favorable conditions (Figure 6).

In flow chamber experiments with *A. oryzae*, Pollack *et al.*[25] followed single hyphae and studied their response to glucose depletion. Similar to our results, they observed secondary growth fueled by carbon recycling, which was morphologically characterized by the formation of hyphae with significantly reduced diameters. For *A. niger* and *A. oryzae*[25,49,50] hyphal diameters were shown to linearly correlate with the specific growth rate, hence the reduction of hyphal diameters reflects the slow rate of secondary growth during the starvation phase. Focusing on non-empty compartments, we analyzed hyphal population dynamics from statistically valid sample sizes for different cultivation time points (Figure 3). Our data showed that older hyphae with larger diameters grown during carbon-sufficient conditions gradually became empty, giving rise to a new population of thinner hyphae. Carbon for this secondary phase of growth might have been liberated from extra- and/or intracellular sources. In agreement with another study of *A. niger*[43], our secretomic data revealed that the relative contribution of lysis was very limited, even under starvation conditions (Table 3). Compared to exponential growth, no relative accumulation of proteins without predicted signal peptide sequences was observed in culture filtrates. However, because these results could also be explained by an equilibrium between proteolytic degradation and leakage of cytoplasmic proteins, it still remains to be shown whether intracellular resources are endogenously recycled by neighboring compartments or first leak into the culture broth where they are subsequently taken up by surviving compartments (Figure 6).

One process known to be important for endogenous recycling of cytoplasmic content in eukaryotes is macroautophagy. In filamentous fungi, it is thought to play an important role in nutrient trafficking along the hyphal network promoting foraging of substrate exploring hyphae and conidiation [51,52]. However, besides endogenous recycling of nutrients, autophagy in general is clearly associated with cell death and is discussed to have protective roles related to the degradation of e.g. damaged mitochondria or unfolded proteins [20,53,54] (Figure 6). It is strongly evident from our transcriptomic data that the induction of autophagic processes is a hallmark of carbon-starved aging fungal cultures. To which extend autophagic processes play a role in the protection against apoptotic/necrotic PCD, endogenous recycling and autophagic PCD remains to be shown in future studies.

The GO enrichment showed a joint downregulation of general protein biosynthesis and secretion pathways during carbon starvation. However, the extracellular accumulation of certain proteins with predicted signal peptide sequences including proteases, glycosyl hydrolases and phospholipases indicates a specification of those pathways which might be related to the emergence of the second population of thin poorly branching hyphae. This phenomenum has been observed, for example, during nitrogen starving surface cultures of *Phanerochaete chrysosporium* for which thin hyphae rather than thick hyphae have been shown to secrete manganese peroxidase [55].

The liberation of carbon from polymers such as fungal cell wall carbohydrates and secreted proteins is indicated by increased expression of glycosyl hydrolases and proteases as well as by increased extracellular protease activity. Strikingly, the major secreted protease PepA [38] was the second most abundant extracellular protein during carbon starvation, which was only excelled by protein levels of the maltose-induced alpha-glucosidae GlaA (An03g06550) secreted during exponential growth. Although transcripts of the ChiB/NagA chitinolytic system accumulated simultaneously during carbon starvation as described previously for *A. nidulans*[16], only NagA could be identified extracellularly in high relative abundances. While the low relative abundance of ChiB in filtrates from day 1 is in agreement with the absence of a predicted signal peptide sequence, it conflicts with results obtained in *A. nidulans*[16], where it was identified as the major extracellular autolytic chitinase. Interestingly, despite its extracellular abundance, also *A. nidulans* ChiB lacks a signal peptide prediction. Whether *A. nidulans* ChiB is released by non-classical secretion or lysis remains to be shown. It is tempting to speculate that cell wall degrading hydrolases lacking a signal peptide sequence are part of the fungal PCD program and accumulate intracellularly in dying compartments to be subsequently released upon cell death for recycling of the remaining hyphal ghost. In view of the natural emerse growth of fungi, this could be a successful strategy for survival - released hydrolases will remain localized to hyphal ghosts and not become diluted as under submerged conditions. Future studies will be necessary to elucidate whether intracellular localization, retention at the cell wall, protein instability or inefficient translation explain the low abundance of ChiB in filtrates of *A. niger*.

Carbon starvation provoked asexual reproduction of *A. niger*, which was clearly evident by the formation of condiospores (Figure 2D) and by expression of respective conidiation-related genes (Table 4). This elaborate developmental program requires liberation and recycling of carbon to proceed in aging batch cultures (Figure 6). Increased heterogeneity and compartmentalization of the hyphal network resulting in empty, cryptically growing and conidiating compartments implies an ordered form of fungal cell death ensuring self-propagation to survive life-threatening starvation conditions. In *A. nidulans* it was shown that disruption of the *flbA* gene, encoding a regulator of G-protein signaling acting upstream of BrlA, resulted in an enhanced autolytic phenotype [8]. Hence, vegetative growth, autolysis and conidiation are closely interwoven processes and future factorial genome-wide transcriptomic studies of wild-type and developmental mutants will allow deconstruction of fungal cell death and its link to developmental processes.

## Conclusions

This study provides a comprehensive description of the carbon starvation response of the filamentous fungus *A. niger* during submerged cultivation. The impact of secondary growth by carbon recycling was indicated by hyphal population dynamics illustrating a gradual transition from old to young hyphae. The induction of autophagic and reproductive processes was clearly evident by major genome-wide transcriptional trends. Hydrolases with strong transcriptional induction during carbon starvation include ChiB, NagA, AgnB, PepA and PepB. Importantly, fragmentation of empty hyphal ghosts was not observed, thus constituting direct evidence that autolysis in aging submerged cultures of *A. niger* is rather initiated by cell death than by hydrolytic weakening and fragmentation of cell walls.

## Methods

### Strain, inoculum and media compositions

Conidial suspensions for inoculation of bioreactor cultures were prepared by growing the *A. niger* laboratory strain N402 (*cspA1* derivative of ATCC9029) [23] on solidified (1.5% agar) complete medium (CM) for three days at 30°C in the dark. Spores were harvested with sterile physiological salt solution (0.9% NaCl) and filtered through Myracloth (Calbiochem, San Diego, CA, USA) to retain mycelial debris and solidified medium. CM contained per liter: 10 g glucose, 6 g NaNO_3_, 1.5 g KH_2_PO_4_, 0.5 g KCl, 0.5 g MgSO_4_·7H_2_O, 1 g casamino acids, 5 g yeast extract and 1 ml trace metal solution. The pH was adjusted to 5.8 with NaOH. The trace metal solution, modified from Vishniac *et al.*[56], contained per liter: 10 g EDTA, 4.4 g ZnSO_4_·7H_2_O, 1.01 g MnCl_2_·4H_2_O, 0.32 g CoCl_2_·6H_2_O, 0.315 g CuSO_4_·5H_2_O, 0.22 g (NH_4_)·6Mo_7_O_24_·4H_2_O, 1.47 g CaCl_2_·2H_2_O and 1 g FeSO_4_·7H_2_O. Minimal medium (MM) for bioreactor cultivations contained per liter: 4.5 g NH_4_Cl, 1.5 g KH_2_PO_4_, 0.5 g KCl, 0.5 g MgSO_4_·7H_2_O and 1 ml trace metal solution. The pH was set to 3 with HCl. After autoclavation, 16 ml of heat-sterilized 50% (w/v) maltose monohydrate solution were added per kg of MM.

### Bioreactor cultivation

#### Inoculation and culture conditions

Batch cultures were performed in 6.6 L BioFlo3000 bioreactors (New Brunswick Scientific) as previously described by Jørgensen *et al.*[11]. Briefly, autoclaved bioreactor vessels were filled with 5 L (kg) sterile MM. During cultivation at 30°C, the controller was set to maintain pH 3 by addition of titrants (2 M NaOH and 1 M HCl). Sterile air was supplied at a rate of 1 Lmin^−1^. Prior to inoculation, 1.5 ml of 10% (w/v) filter-sterilized yeast extract was added to enhance conidial germination. Cultures were inoculated with freshly harvested spore suspensions to give 10^9^ conidia per liter. To reduce the loss of hydrophobic conidia during germination, the stirrer speed was set to 250 rpm and the culture was aerated via the headspace during the first six hours after inoculation. Subsequently, the stirrer speed was increased to 750 rpm, 0.5 ml of polypropyleneglycol P2000 was added as antifoam agent and air was supplied via the sparger.

#### Online measurements

O_2_ and CO_2_ partial pressures of the exhaust gas were analyzed with a Xentra 4100C analyzer (Servomex BV, Netherlands). Dissolved oxygen tension (DOT) and pH were measured electrochemically with autoclavable sensors (Mettler Toledo).

#### Sample preparations

Cultures broth was harvested at regular intervals from batch cultures and mycelial biomass was retained by vacuum filtration using glass microfiber filters (Whatman). Both biomass and filtrate were quickly frozen in liquid nitrogen and subsequently stored at -80°C. Dry biomass concentrations were gravimetrically determined from lyophilized mycelium originating from a known mass of culture broth. Culture broth for microscopic analysis was quickly frozen in liquid nitrogen and stored at -80°C. For LC-MS/MS analysis, 1 ml of Sigmafast protease inhibitor cocktail (S8830, Sigma Aldrich) was added to 30 ml of culture filtrate and BSA was spiked as internal standard (1:10, BSA/total expected protein, w/w) before freezing in liquid nitrogen and storage at -80°C.

### Protease activity assay

Extracellular protease activity measurements were performed similarly to a previously described method by Braaksma *et al.*[57] using N,N-dimethylated BSA as substrate. Measurements were performed in 96 well microtiter plates. 30 *μ*l sample were incubated with 80 *μ*l of 0.5% (w/v) N,N-dimethylated BSA in McIlvaine’s citric acid-phosphate buffer, pH 3, for 30 min at 37°C. Reactions were stopped by addition of 190 *μ*l fresh TNBSA borate buffer solution prepared by adding 50 *μ*l of 5% 2,4,6,-trinitrobenzene sulfonic acid (TNBSA; Pierce) to 10 ml of borate buffer with 0.5 g l^−1^Na_2_*SO*_3_, pH 9.3. TNBSA reacts with primary amines yielding a yellow chromophore that was measured at 405 nm after 10 min. Blank measurements for sample background correction were obtained by incubation of filtrates with citric acid buffer not containing N,N-dimethylated BSA. Non proteolytic release of amines from N,N-dimethylated BSA was assessed by incubation of N,N-dimethylated BSA without filtrate sample. 1 U of protease activity was defined as the activity, which within 1 min under the described incubation conditions produces a hydrolysate with an absorption equal to that of 1 *μ*mol glycine at 405 nm.

### Extracellular protein quantification

Extracellular protein concentrations in culture filtrates were determined using the Quick Start Bradford Protein Assay (Bio-Rad) according to the manufacturer’s instructions.

### Microscopy and image analysis

Microscopic samples were slowly defrosted on ice. For differential interference contrast microscopy (DIC) an Axioplan 2 instrument (Zeiss) with a 100x oil immersion objective was used and micrographs were captured with an DKC-5000 digital camera (Sony). For the automated determination of hyphal diameters, samples were fixed and stained in a single step by mixing them at a 1:1 ratio with Lactophenolblue (Fluka). Sets of 40 micrographs were taken per sample with an DM IL LED (Leica) microscope using a 40x objective and an ICC50 camera (Leica). The microscope and camera settings were optimized to obtain micrographs with strong contrast. To measure hyphal diameters from micrographs of dispersed myclia in an automated manner, the following six-step image analysis algorithm was developed and implemented as a macro for the open source program ImageJ [58]: (1) Convert micrographs to binary images; (2) Copy binary images and outline all objects; (3) Copy binary images and skeletonize all objects; (4) Clean skeletons by removing all intersections; (5) Combine outline and skeleton images; (6) Fragment skeletons and orthogonally measure from the center of each skeleton fragment the distance to the outline.

### RNA extraction, gene chip hybridization and Northern analysis

To minimize the chance of RNA degradation, frozen biomass was directly ground in liquid nitrogen and subsequently total RNA was isolated using the Trizol reagent (Invitrogen) according to the manufacturer’s instructions. Prior to gene chip hybridization, samples were purified on NucleoSpin RNA II columns (Machery-Nagel) including a DNAse I treatment. Lab on chip quality control, labeling, Affymetrix chip (dsmM_ANIGERa_coll511030F) hybridization and scanning were performed at ServiceXS (Leiden, The Netherlands) according to the GeneChip Expression Analysis Technical Manual (Affymetrix inc., 2002). Northern analysis using [*α*^32^P]-dCTP-labelled probes was performed as previously described by Damveld *et al.*[59] using 1.8 *μ*g of RNA per sample. A standard loading control such as 18S rRNA was not used. Equal loading was concluded from smoothly increasing/decreasing time course profiles. Templates for random primer labeling were amplified from genomic DNA of N402 using the following primer pairs: *actA* (An15g00560): 5’-atctcccgtgtcgacatgg-3’ and 5’-gcggtggacgatcgagg-3’; *nagA* (An09g02240): 5’-cccgcgcgaggtatattcac-3’ and 5’-cctgggcgtcagtcagattt-3’; *brlA* (An01g10540): 5’-ggtaacatgtccgatcgcctg-3’ and 5’-gcaactttcctggagggctg-3’.

### Transcriptome data analysis

RNA samples from four cultivation phases were subjected to genome-wide transcriptional profiling: Exponential growth phase, 16 hours (day 1), 60 hours (day 3) and 140 hours (day 6) post carbon depletion. While the expression data for the exponential growth phase was derived from triplicate cultures, expression data for the three post-exponential time points was obtained from duplicate cultures. Transcriptomic data were analyzed with the statistical programming language R [60]. The following packages of the open source and open development project Bioconductor [61] were used: affy [62], affycoretools [63], affyPLM [64] and limma [27]. Affymetrix probe level data was imported from .CEL files and preprocessed with the Robust Multi-array Average (RMA) [65] algorithm as implemented in the affy package. To improve background correction and data normalization, six additional .CEL files corresponding to day 2 and day 8 of carbon limited retentostat cultivations of *A. niger*[11], available at the Gene Expression Omnibus (GEO) database [66] ( http://www.ncbi.nlm.nih.gov/geo/) under accession number: GSE21752, were included in the RMA preprocessing step. Prior to the computation of differentially expressed genes, 65 Affymetrix control probes and 204 probes targeting genetic elements were removed from the expression matrix. For 277 transcripts targeted by multiple probes, mean expression values were calculated from the RMA expression data of all associated probes. Subsequently, RMA expression data for the 13,989 transcripts were analyzed with the limma package comparing day 1, 3 and 6 of carbon starvation with the exponential growth phase. The Benjamini & Hochberg False Discovery Rate (FDR) [67] was controlled at 0.005. Because fold changes are not necessarily related to biological relevance [68,69], a minimal fold change criterion was not applied.

### Annotation enrichment analyses

Enrichment analysis of Gene Ontology (GO) [28] terms was performed using the Fishers exact test Gene Ontology annotation tool (FetGOat: http://www.broadinstitute.org/fetgoat/index.html) [31] applying a critical Benjamini & Hochberg FDR q-value of 0.05. In order to compare and summarize enriched GO terms, we aimed to identify common most-specific GO terms for the sets of up- and downregulated genes, and thus implemented the following algorithm in the Perl programming language: (1) Combine all enriched GO terms from the input sets; (2) Reduce redundancy from higher hierarchy terms by keeping only the most-specific (most-distant) GO terms; (3) For each remaining most-specific GO term, check all parental GO terms, sorted by increasing distance from the corresponding child term, for the presence in the input sets; (4) If a parental GO terms is present in all input sets denote it as common most-specific, if any other equally distant parental terms are present in all input sets, denote them as common most-specific as well and continue with the next most-specific GO term; (5) If none of the parental GO terms are present in all input sets, denote the corresponding most-specific GO term as non-common most-specific; (6) After completing the analysis of all most-specific GO terms, reduce redundancy from the set of common most-specific terms by removing all their parental terms; (7) Output the sets of common most-specific and non-common GO terms. Fishers’ exact test based enrichment analysis of Kyoto Encyclopedia of Genes and Genomes (KEGG) pathway [30] and Pfam domain [29] annotations were performed using in-house developed Perl scripts. The Benjamini & Hochberg FDR was controlled at 0.05. KEGG pathway annotation (file: ang_pathway.list, version from 22.06.2011) for* A. niger* was downloaded from the KEGG homepage ( http://www.genome.jp/kegg/). Pfam domain annotation for *A. niger* was generated by analyzing the predicted proteome of *A. niger* strain CBS 513.88 [1] with the PfamScan Perl script ( ftp://ftp.sanger.ac.uk/pub/databases/Pfam/Tools/PfamScan.tar.gz).

### Secretome analysis

#### Sample pre-treatment

8 ml of 100% (w/v) trichloracetic acid was added to frozen filtrate samples, which were subsequently completely defrosted by shaking at 4°C. Precipitated proteins were spun down and washed twice with ice-cold acetone. Precipitated protein pellets were air-dried, solubilized in 8 M urea (50 *μ*l) and diluted 10x with 100 mM NH_4_HCO_3_. Reduction, alkylation of cysteines and digestion with trypsin were performed according to Thakur *et al.*[70]. Another aliquot of trypsine (2.5 *μ*l 0.25 mg ml^−1^ pH 3) was added after overnight digestion followed by incubation for 3 hours at 37°C to ensure complete digestion. Samples were acidified to 1% formic acid.

#### LC-MS/MS analysis

The protein digests were analyzed in triplicate on an Accela-LTQ-Velos, using a 85 min data dependent LC-MS/MS run, 0-80 min 5-40% B, 80-82 min 40-60% B, 82-83 min 60% B, 83-85 min 5% B (Buffer A 0.1% formic acid in water, buffer B 0.1% formic acid in acetonitrile, both UHPLC grade, Biosolve, Valkenswaard, The Netherlands). Peptide separation was achieved by 25 *μ*l injection on a C18 column (Zorbax SB-C18 2.1x50 mm, Agilent, Santa Clara, CA, USA) using a guard column (Poroshell 300 SB-C3 2.1x12.5 mm, Agilent, Santa Clara, CA, USA) at 50°C and a flow rate of 0.4 ml min^−1^. The data dependent MS method consisted of an enhanced MS scan 300-2000 m/z and MS/MS on the top 10 peaks.

#### Data analysis

The peptide data sets were searched against the *A. niger* database, which was manually modified to contain the sequences of trypsin and BSA, using the Sorcerer 2 Sequest search engine (SageN, San Diego, CA, USA). The search opted for carbamidomethylation (C), oxidation (M) and deamidation (NQ) as variable modifications. The Sequest results were processed using the APEX program [71] according to the author’s description in order to obtain estimates of the protein quantities. Proteins identified with protein probability > 0.9 were considered as significant.

### Microarray data accession number

Microarray data for the post-exponential time points have been made available at the GEO database [66] ( http://www.ncbi.nlm.nih.gov/geo/) under accession number GSE39559. Microarray data from the exponential growth phase were previously made available at the GEO database under accession number GSE21752.

## Competing interests

The authors declare that they have no competing interests.

## Authors’ contributions

BMN performed batch cultivations, protease activity measurements, microscopic and Northern analysis. BMN developed and implemented algorithms for image and GO analysis, conducted transcriptomic data analysis and wrote the manuscript. MA did LC MS/MS analysis. AFJR, VM and TRJ were involved in writing the manuscript. All authors read and approved the final version of the manuscript.

## Supplementary Material

Additional file 1 **Expression data.** Genome-wide transcript profiles, fold changes and FDR q-values. Click here for file

Additional file 2 **GO enrichment.** GO enrichment analysis of genes up-/downregulated at day 1, 3 and 6. Click here for file

Additional file 3 **Pfam enrichment.** Pfam domain enrichment analysis of genes up-/downregulated at day 1, 3 and 6. Click here for file

Additional file 4 **KEGG pathway enrichment.** KEGG pathway enrichment analysis of genes up-/downregulated at day 1, 3 and 6. Click here for file

Additional file 5 **Expression data fumonisin cluster.** Transcript profiles, fold changes and FDR q-values for the fumonisin cluster. Click here for file

Additional file 6 **Expression data PCD genes.** Transcript profiles, fold changes and FDR q-values for putative PCD associated genes. Click here for file

Additional file 7 **Secretome data.** Estimated protein abundances in culture filtrates at day 1, 3 and 6. Click here for file
